# An examination of entrance criteria for international medical graduates (IMGs) into Canadian psychiatry residency programs

**Published:** 2017-02-24

**Authors:** Ashok Soma, Mathew Myatt, Mario McKenna, Soma Ganesa, Ka Wai Leung

**Affiliations:** 1 St. George’s University, New York, US; 2 Alliston Family Health Team, Ontario, Canada; 3 Vancouver Coastal Health, British Columbia, Canada

## Abstract

**Background:**

Although international medical graduates (IMGs) are essential in health care service delivery, a gap exists in the literature about how IMGs are selected into psychiatry residency programs in Canada. The purpose of this study was to identify the relative weight or importance that Canadian program directors (PDs) of psychiatry place on certain selection criteria when matching IMGs into residency programs.

**Methods:**

We electronically distributed a web-based questionnaire to 16 university residency program directors of psychiatry in Canada. Program Directors were asked to rate the importance of 43 selection criteria using 5-point Likert Scales. Criteria were grouped into six domains: academic criteria, extracurricular activities, supporting information, behavioural issues of concern, medical school country, and other education. Mean total values for each set of criteria were calculated and used to create rank orders within each domain.

**Results:**

Eight out of 16 program directors responded. Our analysis indicated that academics and behavioral issues of concern were the most important selection criteria.

**Conclusion:**

Our findings provide valuable insight about the perspectives of Program Directors toward IMGs who apply for psychiatry residency programs in Canada. Further studies are needed to better understand which criteria contribute to IMGs’ performances as psychiatric residents.

## Introduction

International medical graduates (IMGs) are individuals who received their medical training outside of Canada, including immigrant physicians and Canadians who studied abroad.^1^ In Canada, IMGs are essential to healthcare service delivery, making up approximately 25% of the country’s physician work force.^1^,^2^

Historically, the majority of IMGs in Canada emigrated from Britain, Ireland, and South Africa, countries where training and accreditation systems are comparable to Canada’s.^1^ An increasing number of IMGs now come from countries with more pronounced differences in medical training and practice environments, such as Asia, the Middle East, Africa, and Eastern Europe.^1^ Moreover, a 2012 survey by the Canadian Resident Matching Service (CaRMS) estimated that there were 3500 Canadians Studying Medicine Abroad (CSMA).^3^ More than 90% of CSMAs want to pursue postgraduate training in Canada.^1^ Yet in 2012, only 8% (or 274) IMGs were matched through CaRMS into the R-1 match, and a further 3% (or 106) into the second iteration.^3^

Although IMGs are integral to the Canadian physician workforce, research suggests that when compared to their Canadian-trained counterparts, IMGs do not perform as well during their residencies and Royal College specialty examinations.^1^,^2^,^4^ In 2010, an attempt was made to compare IMGs with Canadian Medical Graduates (CMGs) in a BC family practice residency program, by comparing their in-training evaluation reports and Certification in Family Medicine (CCFP) examination results.^2^ The IMG residents compared favorably with their Canadian-trained colleagues on performance during residency training, but not in passing the CCFP examination.^2^ The differences in success rates between IMGs and CMGs for the Royal College of Physicians and Surgeons of Canada (RCPSC) examinations are less pronounced, but they are still evident.^1^ For example, during their first attempts at the primary specialty examination, from 2005 to 2009, 76% of IMGs passed, in contrast to 95% of CMGs.^1^

The process of selecting candidates for residency is often subject to unpredictable factors such as program funding changes and competition within a specialty.^5^,^6^ Program directors (PDs) determine the specific selection criteria, which are used to predict future residency performance and to identify candidates that best fit with the program, for their specialties and their overall level of importance.^6^–^8^ However, as little is known about what is most valued by PDs and selection committees, deans, advisors, and medical students have turned to surveys of PDs to obtain a better understanding of the selection process.^6^,^7^

Despite concerns regarding differences in competency between IMGs and CMGs during postgraduate training,^1^ the literature is lacking in studies that have examined the entrance criteria for IMGs into Canadian Psychiatry Residency Programs available at 14 of the medical schools in Canada.^9^ To our knowledge, a study examining the selection of IMGs into Canadian psychiatry residency programs has not been done. This study aimed to bridge this knowledge gap by describing the criteria used by PDs in selecting IMG applicants into their postgraduate training programs.

## Methods

### Study design

This was a descriptive study that involved the administration of a modified version of the Residency Selection Criteria Questionnaire (RSCQ) to psychiatry PDs in Canada. The RSCQ was originally developed by Wagoner and Suriano^6^ and subsequently modified by Green et al.^10^ This study adapted Green et al.’s version of the RSCQ questionnaire and certain items were amended and added to better fit the Canadian context. A 43-item survey with a 5-point Likert scale was used to examine five domains of residency selection: academic criteria (15 items), extracurricular activities (3 items), supporting information (10 items), issues of concern (8 items) and other factors (8 items).

For the academic criteria domain, PDs were asked to rate the level of importance (1=Unimportant to 5=Critical) for 15 items that related to academic performance, including clerkship narratives, grades for courses required during clerkships and for electives, academic awards, research experience, and examination results. As both Wagnoner and Suriano^6^ and Green et al.^10^ surveyed PDs in the United States, items regarding medical examinations were changed to reflect the requirement necessary for IMGs to pursue postgraduate training in Canada.

The importance of Medical Council of Canada Qualifying Examinations (MCCQE) scores was evaluated using two items – one focused on the scores for Part I of the examination and the other on the score for the complete examination (Parts I and II). Additionally, PDs were specifically asked to evaluate the importance of having done observerships and research with Canadian physicians.

PDs were also asked to evaluate the importance (1=Unimportant to 5=Critical) of experience in three broad categories of extra- and co-curricular activities: leadership roles, community service, and experience in global health.

For other non-academic criteria, PDs also rated the value of having various supporting information in the application (1=Disagree to 5= Agree), including personal statements and curriculum vitaes (CVs), and letters of recommendation and reference. Additionally, they weighted the importance of eight issues of concerns (1=No concern to 5=Very concerned) related to applicants’ personal background and academic records. Examples of these items are: disciplinary action during medical school, extended leave from medicine, and failed exams and courses. PDs were also asked to describe the relative importance (1=Unimportant to 5=Critical) of where IMGs did their medical training and whether they had other graduate degrees.

The questionnaire also contained a section to collect general information about the PDs’ residency programs. In addition, PDs were asked to provide a qualitative response on what they perceived to be the most important factor in selecting IMGS for interviews which, historically, have been a key component for resident selection in psychiatry.^15^

### Participants & survey administration

After institutional ethical approval was obtained, an invitation to complete the RSCQ was delivered via email to 16 Canadian post-graduate PDs of Departments of Psychiatry, from September 1, 2013 to December 31, 2013. The email contained a link to a secure website where directors were requested to complete the RSCQ questionnaire. The questionnaire was completed anonymously and no personal information was recorded or requested of the individuals completing the questionnaire. To maximize the total number of completed surveys, an email reminder was delivered every 30 days, on three separate occasions, after the initial invitation.

### Data analysis

Data from completed questionnaires were downloaded and basic descriptive analysis (frequency, mean, SD, min, max) was used to examine the data. Mean values for each questionnaire item were calculated and used to create rank orders within each domain. All analyses were performed using SPSS V. 17.0 statistical software.

## Results

Fifty percent (8 of 16) of surveyed PDs responded to the questionnaire. Table 1 shows the mean number of positions reported by PDs at their respective institutions for postgraduate year one positions (mean ± SD = 16.1, 6 – 39), positions filled through CaRMS (15.8, 5 – 39), and positions filled by foreign medical school graduates (3.0, 0 – 16).

Table 2 shows the ranking of academic selection criteria by PDs, according to the mean values for each questionnaire item. The mean total score for academic criteria was 43.5 (± 5.5). Among these criteria, clerkship narratives (4.0 ± 0.5) were the highest ranked, followed by grades in required clerkships rotations (3.9 ± 0.6), and scores for the complete MCCQE exam (Part I and II) (3.8 ± 0.7). The least important academic selection criteria were class rank (2.1 ± 1.2), research with Canadian physicians (2.1 ± 0.4) and having taken the USMLE (1.5 ± 1.1).

Table 3 (Appendix A) shows the ranking of non-academic selection criteria by PDs by different domains. The mean total for extra- and co-curricular activities was 8.0 ± 2.2, with leadership (2.9 ± 0.6) and community service (2.9 ± 0.8) scoring the highest importance. The mean total for supporting information was 33.0 (± 5.5), with personal statements (4.0 ± 0.5), letters of recommendation from faculty (4.0 ± 0.8), and the applicant’s CV (3.8 ± 0.5) scoring the highest importance.

The mean total for issues of behavioral concern was 32.4 (± 4.0). The top four criteria that were scored of highest importance were: disciplinary action in medical school (4.9 ± 0.4), absence from medical school for more than three years (4.7 ± 0.5), receiving a failure in a required clinical clerkship (4.4 ± 0.8), and taking extended time to graduate for academic reasons (4.4 ± 0.7).

Mean total score for where IMG applicants attended medical school was 11.8 (± 4.7). Whether their education was from the United Kingdom or Ireland (2.6 ± 1.1), Australia (2.6 ± 1.1), the Caribbean (2.6 ± 1.3), or from another country (2.1 ± 1.0) was of moderately low importance for PDs. The mean total for additional education was 5.4 (± 1.6) with having a PhD (2.1 ± 0.8) ranking highest, followed by MBA (1.6 ± 0.5), or MPH (1.6 ± 0.5).

Seven of the eight responding PDs responded to the question that asked about the most important consideration in selecting IMGs for interviews. Their answers were:

- Genuine interest in psychiatry and graduation from medical school within the past five years
- Good fit with the program
- Medical Council of Canada Evaluating Examination plus composite evaluation of file
- Place of training and time since graduation
- Genuine interest in psychiatry
- Recent, successful clerkship-level work in a Canadian teaching hospital
- Overall application

## Discussion

The present study examined the relative importance that PDs from Canadian psychiatry residency programs have assigned to various selection criteria when matching IMGs into their programs. Although this was a preliminary study, responses from the questionnaires were generally consistent with previously published reports on the most important selection criteria for other residency programs.

The most important selection criterion was issues of behavioral concern and among them, disciplinary action in medical school ranked the highest. Papadakis, et. al.^17^ conducted a case–control study of all University of California, School of Medicine graduates disciplined by the Medical Board of California and found that problematic behavior in medical school was associated with subsequent disciplinary action by a state medical board. However, LeBlanc^16^ pointed out that Papadakis’ study was retrospective and the results only indicated that physicians disciplined by a medical board are significantly more likely to have documentation of unprofessional behavior in their medical school files and not the other direction. Therefore, disciplinary action in medical school is a poor predictor of disciplinary action while practicing as a physician. Therefore, PDs emphasis on disciplinary action as a criterion may not be warranted.

Other important criteria within issues of behavioural concern were receiving a failure in a required clinical clerkship and failing two exams prior to passing. These criteria assessed candidates’ clinical skills and performance and have been shown to be highly valued.^5^–^8^,^10^ Clinical grades have been found to be at least moderately predictive of future residency performance.^7^

The weights assigned to issues of behavioural concern demonstrate how greatly the PDs value academic criteria during the selection process. The issues that were assessed as most important focused on students’ academic performances either directly, such as failure in a required clinical clerkship, or indirectly, such as extended absence from medicine or extended time to graduate for academic reasons. When examined collectively, the emphasis on academic selection criteria over non-academic selection criteria may reflect PDs desire for accurate and objective assessments of the academic and clinical potential of candidates.^6^,^10^

Research and publication experience was ranked very low, which again aligned with the literature.^7^,^8^ Interestingly, this contradicts medical students’ perception that having research experience during medical school is required to successfully obtain their desired residency positions.^10^

Responses for non-academic domains suggest that PDs desire to obtain a well-rounded understanding of IMG applicants as individuals. Having this knowledge will likely help PDs better predict candidates’ future success as psychiatry residents. Letters of support from faculty members, for example, provide valuable insight as to how applicants are perceived.^7^ As with previous studies, PDs in this study valued highly supporting information: personal statements, letters of recommendation from faculty, and CVs were perceived to be the most important.^7^,^8^ In response to an open-ended question in Otero et al.’s study,^8^ PDs identified that the “fit” of candidates in the program was one of the key determinants for selecting radiology residents. Otero et al.’s results were echoed in our study’s open-ended question, as three of the eight PDs explicitly emphasized the importance of IMG applicants being compatible with their programs.

There are several limitations to our study. Preliminary analysis of the findings suggests there is little difference in the selection criteria between IMGs and CMGs; however, our study is limited by a small sample size due to a 50% response rate from Canadian psychiatry program PDs. As voluntary response bias is an inevitable limitation of surveys,^7^,^8^ our findings may not be representative of all psychiatry PDs in the country. Moreover, although the questionnaire responses were anonymous, PDs may have been reluctant to completely acknowledge the level of importance placed on certain RSCQ criteria, such as IMG applicants’ country of origin and location of medical training.

### Conclusion

Our study provides valuable insight into the selection process of IMGs into psychiatry residency programs in Canada, specifically at describing the relative levels of importance that PDs have placed on various criteria. Although this study was descriptive in nature, our findings contribute to a significant gap in the literature by providing important information about the perspectives of PDs in psychiatry. This is much needed given the increasing diversity of applicants for postgraduate training, especially from those who have trained outside of North America. The selection process for residency has thus become more complex, especially in psychiatry, which, out of all medical specialties, has the second highest number of IMGs.^15^

Results of this study may help IMGs learn more about the selection process for Canadian residency programs, assist in strengthening their applications, and help them make better informed decisions about their careers. This information may also be useful for program directors, deans and educators in counseling IMG applicants and in developing their institutional curricula for IMGs.

Lastly, this study highlights the need for future research, specifically which selection criteria best predict residents’ performance. Future studies should obtain a higher response rate, potentially by using a variety of methods to contact PDs. For example, in Green et al.’s study,^10^ questionnaires were sent to PDs electronically and via mail. Of all the questionnaires that reached their destination, 76% (913 of 1201) of the participants mailed their responses, while 24% (288 of 1201) submitted their responses online. To achieve a more comprehensive understanding of the contextual factors at play, it may also be interesting to survey IMGs about how they perceive various selection criteria and examine how their results compare to the PDs’ perceptions.

## Figures and Tables

**Table 1:** Mean Number of Positions Reported by Program Directors

	Mean	SD	Min	Max
Total number of PGY-1 positions	16.1	10.5	6	39
Number of Positions Filled Through CaRMS	15.8	10.8	5	39
Foreign Medical School Graduates (non-Canada/Non-US)	3.0	5.3	0	16

**Table 2:** Program Directors’ Rankings of the Levels of Academic Criteria in Selecting IMGs

Academic Criteria *	Mean	SD	Min	Max
Clerkship Narratives	4.0	0.5	3	5
Grades in Required Clerkships	3.9	0.6	3	5
MCCQE Score (Part I and II)	3.8	0.7	3	5
Grades in Senior Electives of the Specialty	3.6	0.5	3	4
MCCQE Part 1 Score	3.6	0.5	3	4
Electives with Canadian physicians	3.4	0.7	2	4
Grades in Pre-Clinical Courses	3.3	0.7	2	4
Grades in Senior Electives other than that of Specialty	2.8	0.5	2	3
Medical School Academic Awards	2.5	0.5	2	3
Published research while in medical school	2.4	0.5	2	3
Research experience while in medical school	2.4	0.5	2	3
Observerships with Canadian physicians	2.3	0.9	1	3
Class rank	2.1	1.2	1	4
Research with Canadian physicians	2.1	0.4	2	3
Has taken United States Medical Licensing Examination	1.5	1.1	1	4
Total Score	43.5	5.5	37	54

* 1=Unimportant, 2=Somewhat important, 3=Important, 4=Very important, 5=Critical

## Appendix A

**Table 3:** Program Directors’ Rankings of the Levels of Non-Academic Criteria in Selecting IMGs

Extra-curricular Activities*	Mean	SD	Min	Max
Leadership roles	2.9	0.6	2	4
Community Service	2.9	0.8	2	4
Experience in Global Health	2.3	1.0	1	4
Total Score	8.0	2.2	5	11
**Supporting Information****	**Mean**	**SD**	**Min**	**Max**
Personal statements	4.0	0.5	3	5
Letters of recommendation from faculty	4.0	0.8	3	5
Applicant’s curriculum vitae	3.8	0.5	3	4
“Audition electives”	3.6	1.1	2	5
Letters of reference from a non-faculty Canadian Physician	3.4	0.7	2	4
Letters from the Department Chair	3.1	0.8	2	4
Letters of reference from a foreign physician in the intended specialty	3.1	1.0	1	4
Letters of reference from a Canadian physician in another specialty	3.1	0.8	2	4
Usefulness of MSPEs in student ranking	2.9	1.3	1	4
Usefulness of MSPEs to discern underlying problem	2.7	1.3	1	4
Total	33	6	23	40
**Issues of Behavioural Concern****	**Mean**	**SD**	**Min**	**Max**
Disciplinary action in medical school	4.9	0.4	4	5
Has been away from medicine for a period of more than three years	4.7	0.5	4	5
Received a failure in a required Clinical Clerkship	4.4	0.8	3	5
Taken extended time to graduate for academic reasons	4.4	0.7	3	5
Failed exams prior to passing	4.3	0.7	3	5
Received a failure in a pre-clinical Course	3.9	1.0	2	5
Had extended time to graduate for non-academic reasons	3.8	0.5	3	4
Did not participate in any extracurricular activities in medical school	3.3	1.0	2	5
Total Score	32.4	4	26	37
**Medical School Country***	**Mean**	**SD**	**Min**	**Max**
UK/Ireland	2.6	1.1	1	4
Australia	2.6	1.1	1	4
Caribbean	2.6	1.3	1	4
Other	2.2	1.0	1	3
Medical School reputation	2.1	0.9	1	3
Total Score	11.8	4.7	5	19
**Other Education***	**Mean**	**SD**	**Min**	**Max**
PhD	2.1	0.8	1	3
MBA	1.6	0.5	1	2
MPH	1.6	0.5	1	2
Total Score	5.4	1.6	3	7

* 1=Unimportant, 2=Somewhat important, 3=Important, 4=Very important, 5=Critical

** 1=Disagree to 5= Agree

*** 1=No concern to 5=Very concerned
